# Preparation, Mechanical Properties and Strengthening Mechanism of W-Re Alloys: A Review

**DOI:** 10.3390/ma17010102

**Published:** 2023-12-24

**Authors:** Zhenghui Zheng, Chen Lai, Wenyuan Zhou, Ying Wang, Yingxiao Zhang, Jinshu Wang

**Affiliations:** Key Laboratory of Advanced Functional Materials, Education Ministry of China, Materials Science and Engineering, Beijing University of Technology, Beijing 100124, China

**Keywords:** high temperature, tungsten, rhenium, alloy, mechanism, preparation method

## Abstract

W-Re alloys are one of the most important refractory materials with excellent high-temperature performance that were developed to improve the brittleness of tungsten. In the present work, we firstly summarized the research progress on the preparation and strengthening methods of a W-Re alloy. Then, the strengthening mechanisms of the W-Re alloy were discussed, including the influence of Re, solid solution strengthening, second-phase reinforcement and fine-grain strengthening. The results showed that the softening effect of Re was mainly related to the transformation of the preferred slip plane and the introduction of additional d-valence electrons. Some transition elements and refractory metal elements effectively strengthened the W-Re alloy. Carbides can significantly enhance the high-temperature mechanical properties of W-Re alloys, and the reasons are twofold: one is the interaction between carbides and dislocations, and the other is the synergistic strengthening effect between carbides and Re. The objective of this work was to enhance the comprehension on W-Re alloys and provide future research directions for W-Re alloys.

## 1. Introduction

W (tungsten) and W-Re alloys have been widely used in fusion reactor diverters, kinetic energy penetrators, nozzles of rocket engines, welding electrodes, friction welding tools and other components used in high-temperature environments [1,2,3,4,5]. Tungsten and its alloys show many superior properties such as a high melting point, excellent thermal and electrical conductivity, good sputtering resistance and high-temperature strength, which are critical to their application in high-temperature environments [6,7,8,9,10,11]. However, their high brittle-to-ductile transition temperature (DBTT) [12], low-temperature brittleness [10] and recrystallization brittleness [9] are fatal to the service life of tungsten. To solve these problems, one of methods is to alloy it with Re. The introduction of Re into the tungsten matrix not only improves the strength of the alloy but also enhances its ductility at room temperature [13,14]. According to the W-Re binary phase diagram [15], the solubility of Re in W has a maximum value of 37 at.% at 3000 °C and a minimum value of 28 at.% at 1600 °C. When the Re content in a W-Re alloy exceeds 26 wt.%, brittle-phase W_2_Re_3_ will be formed, significantly deteriorating the performance of the alloy. Therefore, the composition of W-Re alloys is usually limited to within 26 wt.%. W-Re alloys are usually fabricated by adding Re with contents of 1, 3, 5, 10, 20, 25 and 26 wt.%. Among them, W-Re alloys with 1~3 wt.% Re have the best high-temperature plasticity, while W-Re alloys with 8~12 wt.% Re possess the best low-temperature plasticity [16]. W-24~26 wt.% Re alloys offer desirable strength and ductility [12]. However, once the content of Re is beyond the solid solubility limit, the Re element is hard to homogenize, leading to Re segregation [17]. Therefore, W-Re alloys with contents of Re in the range of 1~26 wt.% are the main alloys applied in this study.

In order to meet the requirements of friction stir welding tools, rocket nozzles, thermocouples, rotating anode targets, thermoelectric energy converters, etc., [18,19,20], a lot of research has been focused on improving the strength and the strengthening mechanism of high-temperature of W-Re alloys. But to the best of our knowledge, there have been no reviews on W-Re alloys. Herein, this paper systematically summarizes the preparation methods for different W-Re alloys, the properties and strengthening approaches of W-Re alloys and their strengthening mechanism, including the role of Re in the tungsten matrix, solid solution strengthening, second-phase strengthening and the grain refinement strengthening mechanism. Finally, this review gives a systematical summary on W-Re alloys and offers prospects for researchers on enhancing W-Re alloy performance.

## 2. Preparation Methods of W-Re Alloys

The fabrication of W-Re alloys is full of challenges due to their high melting points. Smelting and powder metallurgy are two common methods for fabricating W-Re alloy. Clarifying the features of the two preparation methods of W-Re alloys is essential to improve their mechanical properties.

### 2.1. Smelting Methods

Arc smelting and electron beam smelting have been used to prepare W-Re alloys [21]. Arc melting is a method of melting materials by using an electric arc. Kang et al. prepared a W-Re-SiC alloy by arc melting, which possessed a high strain capacity (37.1%) at room temperature [22]. The grain sizes of single-crystal W-Re-Mo alloys fabricated by arc melting can reach 1~4 mm [23]. Large-sized W-Re alloys doped with a second phase such as HfC and ZrC were obtained by using the arc melting method [24,25,26]. However, the molten state with arc melting is short, which makes the residual gas in the billet difficult to eliminate and leads to the formation of voids [22,23,27].

Electron beam melting is a metallurgical technique for melting materials by using a high-velocity electron beam, the kinetic energy of which is converted to thermal energy by bombarding the target materials. The impurity of materials prepared by beam smelting is low. Recently, selective laser sintering smelting has been developed, which utilizes a high-energy-density laser beam (10^4^~10^5^ W/cm^2^) to achieve rapid melting, diffusion, cooling and solidification [16,28,29,30]. Electron beam melting has a high production efficiency and allows for the fabrication of components with complex shapes. Tungsten collimators with complex structures were successfully fabricated by the selective laser melting technique [31]. Zhou et al. analyzed the spheroidization of tungsten crystal during selective laser melting [32]. Dou et al. studied the parameters of selective laser melting on the microstructure and properties of a W-Re alloy [16]. However, selective laser melting makes the powder prone to oxidation, leading to a low relative density of the final product [28]. For example, the relative densities of tungsten bulk fabricated by Deprez et al., Zhou et al. and Dou et al. were 89.9%, 82.9% and 96.1%, respectively [16,31,32]. Therefore, increasing the relative density of the billets manufactured by selective laser melting is crucial to expanding their applications.

The crystals of W-Re alloys prepared by the above two methods are columnar. In order to refine the grains and increase the relative density of W-Re alloys, plastic deformation and annealing treatments are indispensable. However, the plastic deformation processing increases the cost of W-Re alloys [33], which impedes the utilization of these smelting methods in the preparation of refractory metals.

### 2.2. Powder Metallurgy

**Figure 1:**
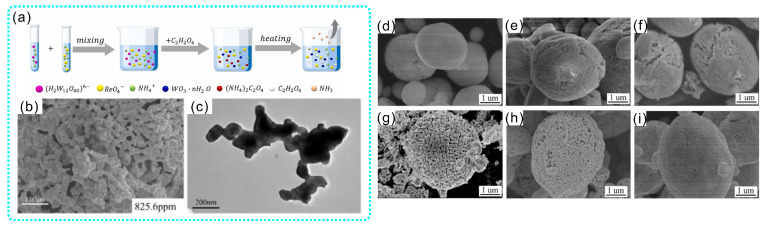
(**a**) Schematic diagram of the synthesis process of W-Re precursor powders. (**b**) Microstructure of W-10Re precursor powder reduced at 1000 °C, and (**c**) the powder corresponds to TEM images [34]. Microstructure of W-75Re precursor powders prepared by the spray-drying process with solution concentrations of (**d**) 20 g/L, (**e**) 40 g/L and (**f**) 60 g/L, and the microstructure of powders with solution concentrations of 20 g/L reduced at (**g**) 350 °C for 2 h followed by 950 °C for 2 h, (**h**) 450 °C for 2 h followed by 950 °C for 2 h and (**i**) 550 °C for 2 h followed by 950 °C for 2 h [35].

Compared with the above smelting methods, the powder metallurgy method is a low-cost, repeatable and simple process that is widely used in the preparation of refractory metals [33]. The processes of preparing W-Re alloys by using powder metallurgy includes the preparation of the composite powder, compression molding and high-temperature sintering. W-Re composite powders are usually prepared via mechanical alloying (MA) and wet chemical methods. The advantages of mechanical alloying are its low cost and feasible operation. In addition, the grain size of the composite powder is finer after mechanical alloying [12]. However, mechanical alloying not only introduces impurities but also increases the amount of powder agglomeration. Ammonium metatungstate and ammonium perrhenate were used as raw materials to prepare precursor powder for the wet chemical method. Subsequently, a W-Re alloy powder was obtained after reducing in a hydrogen atmosphere, the elements distribution of which was uniform. The morphology of W-Re alloy powder is closely related to the preparation method of the precursor. Xu et al. prepared W-10Re alloy powders via the wet chemical method, as shown in Figure 1a [34]. The morphology of the alloy powders after hydrogen reduction is shown in Figure 1b,c. It can be seen that the powders exhibited a coral-like morphology. Lai et al. prepared a spherical W-Re precursor powder via the spray-drying method [35]. The microstructure of the precursor powder is indicated in Figure 1d–f [35]. By regulating the reduction parameters, spherical/quasi-spherical powders with pores were fabricated (Figure 1g–i).

The sintering methods of powder metallurgy include pressureless sintering and pressure sintering. Pressureless sintering includes vacuum sintering and atmospheric sintering. Hydrogen is the most commonly used gas in atmospheric sintering, and this method is used to eliminate the residual impurities in the billet. A W-Re alloy with the relative density of 98% was prepared by low-temperature sintering under a hydrogen atmosphere, during which the sintering temperature ranged from 1100 to 1300 °C [36]. Vacuum sintering is another commonly used pressureless sintering method. Zhang et al. prepared a ZrC-doped W-Re alloy by vacuum sintering. The results indicated that the recrystallization temperature of the W-Re-ZrC alloy was 1600 °C after rolling [37]. Due to the positive correlation between the relative density and the sintering temperature, the grain size of W-Re alloys is difficult to control. Though W-Re alloys can be obtained by pressureless sintering at relatively low temperatures [36], the time (about 10 h) required for the sintering temperature is long. W-Re alloys were also prepared via the spark plasma sintering (SPS) method. By applying simultaneous pressure and resistive pulse heating, rapid densification and sintering were achieved at relatively low temperatures while slowing the rate of grain growth. Oda et al. successfully fabricated a W-10 wt.% Re alloy with a grain size of 0.45 μm and a relative density of 99% at 1600 °C by spark plasma sintering [38]. However, spark plasma sintering is not only low efficient but also the sample size is limited.

## 3. Properties and Strengthening Methods of W-Re Alloy

### 3.1. Brittleness of Tungsten

The lack of inherent close-packed planes and the weak bonding of the grain boundaries lead to the low toughness and ductility of tungsten [39]. The ductility of tungsten depends on the migration of its non-planar 1/2<111> screw dislocations [40]. The dislocation cores of tungsten are distributed on three {110} planes of the <111> zone, resulting in a higher Peierls stress [41], which refers to the stress required for dislocation slip at 0 K. The spread of the 1/2<111> dislocation cores forms a non-planar structure, which leads the dislocation being difficult to move along the slip systems in tungsten [42], resulting in the brittleness of tungsten. In addition, the weak binding force between the grain boundaries increases the brittleness of tungsten and consequently leads to grain boundary fractures, which have been demonstrated by experimental [43] and theoretical results [44]. It is generally believed that impurities, such as potassium (K) and nickel (Ni), present on the grain boundaries decrease the bonding strength of the grains and increase the number of intergranular fractures [45,46,47]. Though impurities are scarcely observed at the grain boundaries of high-purity tungsten, intergranular fracturing is also inevitable [43]. The intergranular fracturing mechanism of tungsten is still unclear.

Methods of mechanical processing, alloying and grain size refinement have been developed to enhance the ductility of W. The ductility of sintered tungsten is increased after rolling or extrusion [48]. The improvement in the ductility is attributed to the high dislocation density and laminated structure in tungsten after its deformation [39]. However, the plasticity of tungsten degrades rapidly after annealing and recrystallization at high temperatures [49,50]. Reier et al. found that deformed tungsten foils exhibited good ductility at room temperature. Nevertheless, the texture and dislocations were reduced after high-temperature annealing, resulting in a decrease in the ductility and an increase in the ductile–brittle transition temperature (DBTT) [49].

Geach and Hughes reported that a W-35 at.% Re alloy, which was prepared by a combination of arc melting and secondary processing, exhibited a higher ductility than pure tungsten [39]. However, distinguishing the effects of mechanical processing and Re on the ductility improvement in tungsten is difficult. Mutoh et al. compared the toughness of pure tungsten and W-Re alloy without secondary processing [51]. It was revealed that the ductility and the toughness of tungsten could be improved by Re. Moreover, the toughness of the W-Re alloy increased significantly with the increase in the Re content [51]. Subsequent studies have found that Re alloying reduces the Peierls stress and increases the number of sliding planes, which contribute to the ductility of tungsten [52].

The ductility of W-Re alloys is sensitive to the grain size. A small grain size provides long-range barriers to dislocations, thereby reducing the strain rate sensitivity of W-Re alloys [53]. However, the deformation mechanism of W-Re alloys varies with the grain size when the temperature is higher than 0.2 T_m_ (melting point of tungsten). The deformation behavior of coarse-grained W-Re alloys is controlled by dislocation–dislocation interactions [54,55], while it is controlled by dislocation–grain boundary interactions for ultrafine-grained W-Re alloys [56,57]. This transition in the plastic deformation behavior is attributed to the thermally activated accommodation of lattice dislocations within the grain boundaries with increasing temperatures [58,59].

### 3.2. Influence of Re on the Microstructure and Properties of W

The dislocation structure has a great influence on the brittleness of tungsten. Clarifying the influence of Re on the dislocations is crucial to understanding the plastic deformation behavior of tungsten alloys. It has been found that d-valence electrons play an important role in the movement of dislocations in tungsten and other high-melting-point metals with body-centered-cube (BCC) crystal structures [40,60,61,62,63]. For tungsten alloys, a large number of d valence electrons enhance the double kink nucleation of screw dislocations, promote dislocation migration and achieve solid solution softening. On the contrary, a lack of d valence electrons leads to hardening and embrittlement [61,62]. Extra d-valence electrons could be introduced by adding a small amount of Re (1–8 at.%), thus providing a softening effect [64].

**Figure 2:**
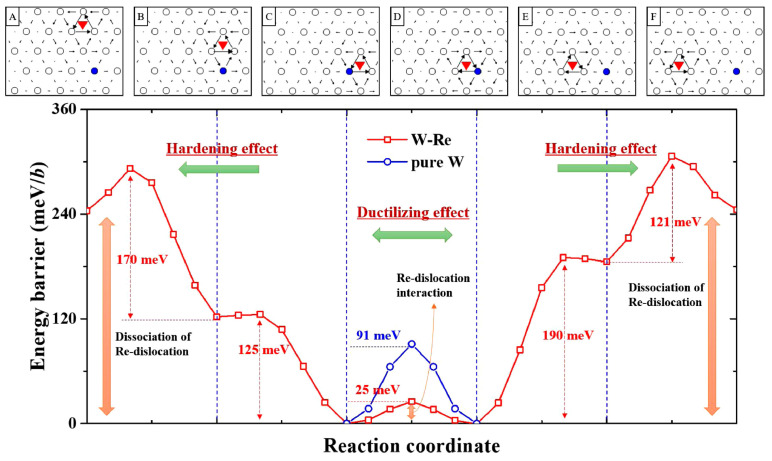
Energy difference between W-Re and pure tungsten with or without Re atoms during the motion of 1/2<111> screw dislocation [68]. The figures (**A**–**F**) represent the interaction process between dislocations and Re atoms during dislocation motion. The downward red triangle represents the Burgers vector of 1/2<111> screw dislocation core, and the blue dots represent single Re atom.

The plastic deformation of BCC metals is related to non-planar screw dislocations, which induce high lattice friction during diffusion [40]. It is worth noting that there are many types of dislocation cores in BCC structures [65,66]. The symmetry of the dislocation core affects the slip plane and consequently affects the macroscopic plastic deformation behavior of alloys. Romaner et al. found that, after alloying with Re, the symmetric screw dislocation core in tungsten transformed into an asymmetric one. In addition, the slip system of the dislocation changed from {110} to {112} [52]. The latter had six more slip planes than the former. Consequently, the Peierls stress of W-25 at.% Re alloy decreased from 2.49 GPa for pure tungsten to 1.84 GPa [40,52,67]. Li et al. studied the influence of Re on the Peierls energy in tungsten [68]. Figure 2 shows the energy barrier of the migration of the screw dislocation with and without Re atoms. When the screw dislocation approaches Re atom (as shown in Figure 2 from A to B, from B to C, from F to E and from E to D), the energy barriers are 49 meV/b, 3 meV/b, 61 meV/b and 5 meV/b, respectively, much lower than the 91 meV/b found in pure W. However, when the screw dislocation moves away from Re (as shown in Figure 2 from C to B to A and from D to E to F), the energy barriers increase to 121–190 meV/b. Interestingly, the energy barrier is a low as 25 meV/b when the screw dislocation migrates from C to D, indicating that Re effectively reduces the shear resistance of screw dislocation migration in tungsten. It promotes the movement of screw dislocation during plastic deformation. These results are consistent with the findings of Medvedeva and Qian, who found that the presence of Re reduced the generalized stacking fault energy (GSFE) and shear resistance of <111>{110} in W, leading to an increase in the migration rate of 1/2<111> screw dislocations [62,69,70].

In addition, the Re content has an impact on the thermal diffusivity of tungsten. The thermal diffusion of tungsten atoms in W-Re alloys can be represented by Equation (1) [71,72,73,74,75,76].

*k* = *α*∙*C_p_*∙*d* (1)

where *k* is the thermal conductivity, *α* is the thermal diffusivity, *C_p_* is the specific heat and *d* is the mass density. The thermal conductivity is affected by the microstructure of the material. The heat energy of the material can be calculated with electronic and phonon conduction [77]. Compared with the contribution of phonons to the heat conduction in silver and aluminum (6 W/mk for aluminum and 4 W/mk for silver), the contribution of phonon thermal conductivity to that in tungsten is much higher (46 W/mk), which accounts for about 33% of the total thermal conductivity [78]. A physical model considering the influence of phonons was used to predict the thermal conductivity of W-Re alloys (with Re contents ranging from 1 wt.% to 25 wt.%) in the temperature range of 300~1200 K [77]. It was indicated that a high concentration of Re greatly reduced the thermal conductivity of the W-Re alloys [77]. Huang et al. studied the effects of temperature and the aggregation morphology of Re on the thermal conductivity of W-Re alloys by using molecular dynamics simulations [79]. The results showed that as the temperature increased, the phonon scattering increased while the thermal conductivity decreased. The thermal conductivity of the W-Re alloy with spherical Re clusters was the highest [79].

Thermal expansion is also an important property in W-Re alloys. Density functional theory (DFT) is a method that allows for the calculation of the ground state energy and properties of a system through the electron density. This method can be used to obtain the ground-state electronic structure, geometric parameters, mechanical properties, thermodynamic properties and other material characteristics of solid materials [80]. Zhou et al. used the DFT method to calculate the effect of Re content (3.125/6.25/9.375 at.%) on the thermal expansion properties of W-Re alloys [81]. The results showed that the heat capacity at constant pressure (*C_p_*) and heat capacity at constant volume (*C_v_*) gradually decrease in W-Re alloys with the increase in the Re content, resulting in an increase in the thermal expansion coefficient. However, the potential function used in Zhou et al.’s work cannot accurately predict properties such as the kinetic processes and defect behavior at high temperatures [82]. It only agrees well with the previous calculation results below 500 K [83]. Dengg et al. used a quasi-harmonic approximation to calculate the thermal expansion coefficient of W-Re alloys with 0–50 at.% Re at 2400 K. The results showed that the increase in the thermal expansion coefficient when the Re content exceeded 12 at.% was due to the softening of strong phonons at smaller wavelengths accompanied by an increase in the Grüneisen parameter.

### 3.3. Solid Solution Strengthening of Elements

The solid solution method is one of the effective methods to improve the ductility of W. It has been widely demonstrated that Re helps to improve the ductility of tungsten [62,84,85]. However, Re induces the formation of intermetallic phases or clusters, which leads to a hardening effect in tungsten [86,87,88,89]. Therefore, some elements are added to inhibit the formation of intermetallic phases and improve the ductility of W.

Transition elements have been suggested to improve the ductility of W. It has been reported that a small amount of transition elements can be used as activators and to reduce the sintering temperature to 1200–1500 °C, thereby slowing down the growth of grains [90]. Kishore et al. studied the influence of lanthanum (La) and copper (Cu) on the mechanical properties of W-Re alloys by using DFT [7]. The results showed that the W_0.76_Re_0.14_La_0.10_ alloy is mechanical instability. Additionally, it was found that W_0.81_Re_0.14_Cu_0.05_ alloy possessed a moderate hardness, bulk modulus, shear modulus and Young’s modulus. Skotnicova et al. compared the yield strength of single-crystal W, W-2Re and W-1Mo-1Re. It was found that the yield strengths of the W-2Re and W-1Mo-1Re were 22% and 28% lower than that of W, respectively [23]. Liu et al. investigated the influence of Ta on the microstructure and properties of W-10Re alloy [9]. The results showed that Ta can effectively refine the grain size. When the Ta content reached 30 wt.%, the grain size of the W-Re-Ta alloy decreased from 17.3 to 6.7 μm, while the yield strength was increased by 183%, reaching 1248.8 MPa. However, the amount of oxides in the matrix increased with the increase in the Ta content, thus accelerating transgranular fracturing [9]. Xiao et al. prepared a single-phase W-Re-Ta alloy by using the selective electron beam melting method [29] and analyzed their distribution in different directions. The results showed that the grain size, Vickers hardness and compressive strength varied along the build direction. For example, the hardness and grain size in the XOZ section were 668 HV and 30.9 μm, respectively, while in the XOY section, they were 560 HV and 29.5 μm, respectively.

Moreover, the effect of the alloying order needs to be considered, especially the structural order, which greatly affects the ductility of tungsten solid solutions [91]. Recently, Bao et al. studied the effect of Mo and the alloying order on the mechanical properties of W-Re using DFT [91]. The results showed that the short-range ordered (SRO) W-Re alloy exhibited a better ductility. The SRO structure of the Mo-doped W-Re alloy promoted the strengthening of the coherent bonding between the W, Mo and Re, showing a higher stability and strength. This indicates the importance of the alloying order in ternary W-Re alloys.

### 3.4. Second-Phase Reinforcement

The high-temperature strength of tungsten decreases with increasing the temperature [92]. The addition of Re scarcely improves the high-temperature strength of tungsten. Therefore, the high-temperature strength of W-Re alloys can be enhanced by a second phase.

HfC possesses the highest melting point and the lowest saturation vapor pressure among the potential second-phase candidates, and it has an excellent high-temperature thermodynamic stability. Therefore, it has been used to enhance the mechanical properties of W-Re alloys [93]. Li et al. prepared W-3Re alloys with HfC contents of 0, 0.5, 1, 5 and 10 wt.% via the SPS method. They found that adding 10 wt.% HfC increased the hardness and compressive strength of the W-Re alloy by 92.5% and 286%, respectively [8]. They further suggested that grain refinement, Orowan strengthening and interface thermal mismatching were the main mechanisms for the strengthening effect of HfC [8]. Yun et al. studied the tensile strength of W-24Re-HfC and W-4Re-HfC wires. The results showed that the tensile strength of the W-24Re-HfC wire at room temperature was 3250 MPa, which was slightly higher than that of the W-4Re-HfC wire (3160 MPa). However, the W-4Re-HfC wire exhibited a higher tensile strength and stress fracture strength above 1366 K [94]. It was indicated that the Re content in W-Re-HfC alloys plays an important role in their high-temperature performance. Iqbal et al. studied the effects of HfC on the frictional performance of a W-25Re alloy. The results showed that the average coefficient of friction (COF) and wear rate of the W-25Re significantly decreased after adding the HfC, effectively improving the tribological performance of the W-25Re [95]. Though the HfC improved the high-temperature strength of the W-25Re, it also reduced the toughness of the W-25Re, ultimately leading to intergranular fracturing and wear failure [96].

ZrC has a high melting point, high formation energy and high thermal stability, and it is considered as a good potential candidate for use in the second phase [25,97]. Yang et al. studied the high-temperature tensile behavior of W-1Re-0.5ZrC. The results showed that the ductile–brittle transition temperature of the W-1Re-0.5ZrC was about 400–500 °C, which was lower than that of pure tungsten by 200 °C. The low ductile–brittle transition temperature of the W-1Re-0.5ZrC resulted in an elongation of over 30% at 600 °C, which was approximately twice as much as that of pure tungsten at 700 °C [97]. Zhang et al. investigated the recrystallization behavior of W-1Re-0.5ZrC after hot rolling. It was found that the recrystallization temperature of the W-1Re-0.5ZrC was about 1600–1700 °C, which was significantly higher than that of pure W (1200 °C) [37]. Miao et al. observed that the microstructure and hardness of W-10Re-0.5ZrC changed little after annealing at 1500 °C for 1 h. The tensile strength and elongation behavior of W-10Re-0.5ZrC at 300 °C were 818 MPa and 8.1%, respectively [96]. Recently, Miao et al. studied the effects of ZrC on the properties of W-25Re-0.3ZrC. The results showed that the elongation behavior of W-25Re-0.3ZrC was only 16.1% at room temperature, which was lower than the 31.6% of W-25Re. The W-25Re-0.3ZrC maintained a high elongation behavior of 12.9% after annealing at 1600 °C for 1 h. The toughness of the W-25Re decreased significantly after annealing at 1600 °C for 1 h. These studies suggest that low-angle grain boundaries and dislocation contribute to the improvement in the plasticity of W-Re alloys. ZrC helps to keep the stabilities of the low-angle grain boundaries and dislocation at high temperatures. Thereby, the annealing-induced brittleness of W-Re is suppressed [98].

**Figure 3:**
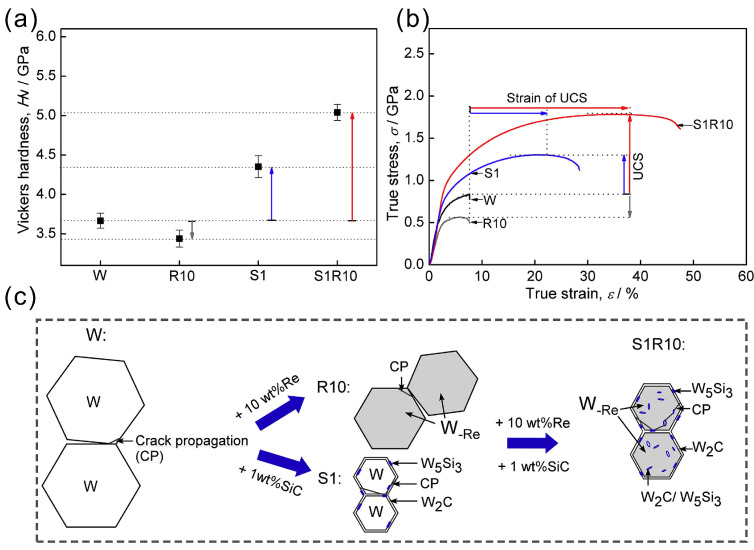
(**a**) Vickers hardness, (**b**) stress–strain curves and (**c**) schematic diagrams of compressive fracture mechanisms of W, W-10Re, W-1SiC and W-10Re-1SiC [22]. The symbol “UCS” in (**b**) represents “Ultimate Compressive Strength”. The symbol “W”, “R10”, “S1”,“S1R10” in (**a**–**c**) represents “W”, “W-10Re”, “W-1SiC”, “W-10Re-1SiC”, respectively.

In addition to refractory metal carbides, SiC is also used as a toughening phase to strengthen W-Re alloys and is generated in situ at high temperatures. Lee’s studies showed that the bridging effect induced by W_5_Si_3_ significantly improved the flexural strength of W-SiC at room temperature [99]. Kang et al. analyzed the synergistic effect of SiC and Re on the hardness and toughness and their mechanisms, as shown in Figure 3 [22]. Figure 3a shows that the addition of SiC effectively increases the hardness of pure W. Moreover, the hardness of tungsten can be further increased by the synergistic effect of SiC and Re. A similar trend is also observed in the stress–strain curve of W-Re alloy doped with SiC, as shown in Figure 3b. These results indicate that the synergistic effect of Re and SiC greatly contributes to the mechanical properties of W. The mechanism is shown in Figure 3c. The synergistic reinforcement originates from the toughening effect of Re on the matrix and the particle strengthening effect of SiC, both of which synergistically interact with long-range particles and dislocations. Simultaneously adding Re and SiC can toughen the matrix and strengthen the internal particles [22]. In addition, ThO_2_, the highest-melting-point oxide (3473 K), was used to enhance the high-temperature performance of W-Re alloys. Luo et al. studied the high-temperature tensile properties of W-26Re-1ThO_2_. It was found that the ThO_2_ increased the temperature sensitivity and strain hardening rate of the W-26Re at high temperatures [100]. However, due to the large size of ThO_2_ particles and the agglomeration of the grain boundaries of ThO_2_, the high-temperature strengthening effect of ThO_2_ is limited.

The second phase is usually added in the form of carbide. Recently, second phases have been prepared by in situ synthesis during the sintering process [101,102], which generally involves mixing refractory metal hydrides with carbon powder and generating carbides through reaction during sintering. Cheng et al. prepared a W-ZrC alloy by mixing ZrH_2_ powder, carbon powder and tungsten powder [101]. The results showed that small-sized ZrC particles (approximately 20 nm) were formed inside the grains, along with ZrO_2_ particles larger than 100 nm. Liu et al. prepared a W-Re-HfC alloy using W-Re powder, HfH_2_ powder and carbon powder as the raw materials [102]. The results indicated that the composite material consisted of a W-Re matrix, HfO_2_ and HfC. It was found that the particle size of the HfO_2_ exceeded 1 μm, while the particle size of the HfC was approximately 100 nm. The possible mechanism for in situ formation of carbides is the decomposition of hydrides at low temperatures (approximately 500~700 °C), where refractory metals dissolve into the matrix at sintering temperatures and eventually precipitate as stable carbide particles in combination with carbon atoms [101].

It is worth noting that the formation of carbides in situ is usually accompanied by the generation of large-sized oxides. There are several reasons for the formation of large-sized oxide particles. First, both the groups used the powder metallurgy process of ball-milling, cold isostatic pressing, sintering and forging to prepare the samples [101,102]. Even with a protective atmosphere, a prolonged high-energy ball milling time (24 h) may lead to the decarburization of carbides and an increase in the oxygen content in the powder [102]. Second, carrying out the forging process in air after heating the samples to a high temperature of 1600 °C will also promote the decarburization of carbides [101]. Finally, the oxidation of hydrides in the raw materials [102] or the absorption of oxygen by the refractory metal at the grain boundaries during the formation of carbides [101] can lead to large-sized oxides.

W-Re alloys have been widely used in high-temperature environments due to their excellent high-temperature strength and hardness. As we know, the mechanical properties of W-Re alloys can be significantly improved by a second phase. Therefore, the structures and properties of second-phase-doped W-Re alloys used in high-temperature environments are analyzed below.

**Figure 4:**
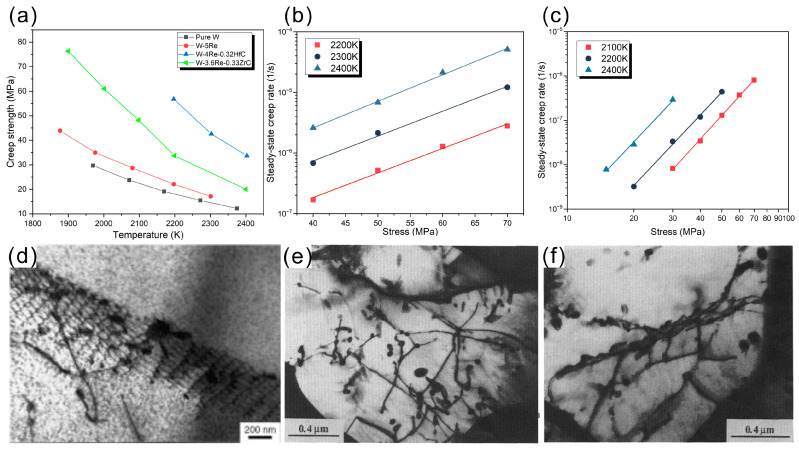
(**a**) Creep strength of different W-Re-carbide systems at a creep rate of 10^−6^/s in the temperature range from 1600 K to 2400 K [25,104]. (**b**,**c**) The linear relationship between steady-state creep rate and stress for W-4Re-0.32HfC and W-3.6Re-0.33ZrC, respectively [25,105]. Subgrain structure images from (**d**) W-4Re-0.32HfC and (**e**,**f**) W-3.6Re-0.33ZrC tests at 1900 K and 2200 K, respectively [25,104].

Creep is an inevitable process of plastic deformation that occurs during operation at high temperatures [103]. It is defined as the time-dependent plastic deformation of materials under high-temperature and low-stress conditions. The creep behavior of materials should be considered during the material design, otherwise the lifetime of the materials will fall short of expectations [103]. The creep strength is an important parameter for estimating the long-term creep performance of materials [104]. Figure 4a illustrates the effects of a second phase on the creep strength of tungsten and tungsten alloys, as reported in the literature [25,104,105]. It can be seen that pure W has the lowest creep strength at different temperatures, and the addition of a small amount of Re slightly improves the creep strength of W-Re alloys. The addition of Re can increase the creep activation energy by 28 kJ/mol [106] and provide solid solution strengthening, resulting in the higher creep strength of W-Re alloys than pure W. Moreover, the creep strength of W-Re alloys is significantly improved after adding ZrC. However, the creep strength of the W, W-Re and W-Re-ZrC systems converges to a saturation value as the temperature increases, indicating that the strengthening effect of ZrC on W-Re alloys decreases with increasing temperature [25]. The decrease in the strengthening effect is attributed to the evolution of dislocation under elevated temperatures, as shown in Figure 4e,f. Dislocation entanglement and dislocation pinning are observed in ZrC-doped W-Re alloys at 1900 K. However, the dislocation entanglement disappears and a dislocation network/wall is present at 2200 K. Compared with ZrC-doped W-Re alloys, HfC-doped W-Re alloys exhibit a higher creep strength, which is attributed to the obstruction of the dislocations by the subgrain structure (shown in Figure 4d) [104]. The relationships between the steady-state creep rate and temperature of W-Re-ZrC and W-Re-HfC alloys are indicated at Figure 4b,c, respectively [25,104]. It can be seen that the relationship between the stress and steady-state creep rate is linear in the two samples. The small changes in the slope of the lines indicate that the stress exponents of W-Re alloys with ZrC and HfC additions are insensitive to temperature [104].

**Figure 5:**
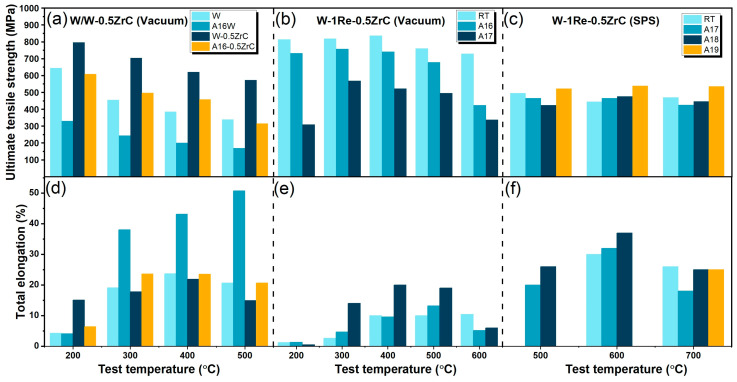
Comparison of ultimate tensile strength and total elongation in the test temperature range from 200 to 700 °C in different W-Re-ZrC alloys. (**a**,**d**) W and W-ZrC with or without annealing at 1600 °C [107]; (**b**,**e**) W-1Re-0.5 ZrC sintering in vacuum [37]; (**c**,**f**) W-1Re-0.5 ZrC prepared by SPS technology [97]. A15 to A19 in the figure indicate the samples annealed at 1500 °C, 1600 °C, 1700 °C, 1800 °C and 1900 °C, respectively.

The high-temperature tensile performance is important in evaluating the plastic deformation capability of materials at high temperatures. Tungsten and its alloys have attracted much attention due to their excellent high-temperature strength. Deng et al. analyzed the effect of ZrC on the ultimate tensile strength and total elongation behavior of hot-rolled tungsten prepared by vacuum sintering at high temperatures, as shown in Figure 5a,b [107]. It is evident that the W-ZrC alloy, without undergoing the annealing process, exhibited the highest ultimate tensile strength across all the tested temperatures. It is also noted that after annealing, the ultimate tensile strength of all the samples decreased while the total elongation increased. The elongation rate of the pure tungsten increased most significantly, from 4.1% to 50.7%. Zhang et al. studied the synergistic effect of ZrC and Re on the high-temperature tensile performance of hot-rolled tungsten alloys prepared by vacuum sintering, as shown in Figure 5b,e [37]. Consistent with the results of Deng et al., the tensile strength of the tungsten alloys decreased after annealing at high temperatures. The standard deviations of the tensile strength for the W-Re-ZrC and the W-ZrC alloys were 39.9 and 84.8, respectively. It is indicated that the thermal stability of the W-ZrC alloy was improved by the addition of Re. In addition, the tensile strength decreased with the increase in the annealing temperature. However, the total elongation increased with the increase in the annealing temperature. Yang et al. studied the high-temperature tensile performance of a W-Re-ZrC alloy prepared via the SPS method after annealing at 1800 to 1900 °C for 2 h, as shown in Figure 5c,f [97]. It can be seen that annealing had little effect on the tensile strength of the samples obtained via the SPS method. The rolling process induced a high dislocation density in the specimens, which subsequently enhanced the high-temperature tensile strength. However, the high-density dislocation disappeared after the annealing treatment, resulting in a decrease in the tensile strength and tensile strength fluctuation.

Erosion and thermal shocking are challenges that W-Re alloys must face up to when used as certain high-temperature components such as rotating anode tubes and rocket nozzles [19,30,108]. Siller et al. studied the surface structural damage of W-10 wt.%Re by using an electron beam welding machine under thermal shocking [108]. The results showed that the defects on the surface of the samples included slip bands, grain boundary bulging, cracks, pitting and thermal grooves after the thermal shock cycles. The columnar grain structure decreased the surface pitting of the grains but increased the surface expansion. The addition of HfC not only refined the recrystallized grains but also impeded the surface expansion and pitting [108]. Li et al. investigated the influence of HfC on the ablative resistance of W-Re alloys via the oxyacetylene erosion method [19]. The results showed that adding 0.5 wt.% HfC significantly decreased the ablation rate of the W-3Re from 6.67 μm/s to 2.93 μm/s. However, the linear ablation rate decreased to 2.23 μm/s when the content of HfC was increased to 5.0 wt.%. They believed that the ablation mechanism of the W-3Re-xHfC was attributed to the thermal–chemical oxidation of the W-Re matrix and HfC [19]. Yang et al. studied the ablation resistance of pure tungsten and W-Re alloys. The results showed that the cracking in the tungsten was greatly suppressed even when the content of Re was low (1 wt.%, 5 wt.%), while the ablation resistance of the pure tungsten changed little. The cracking was significantly suppressed when the content of Re was high (25 wt.%). However, the ablation threshold of the W-25Re alloy was low, leading to a large ablation depth and a high rate [30].

Friction stir welding (FSW) is a novel solid-state welding technique. This method employs a non-consumable rotating head to exert pressure against the mating surface of the workpiece. By means of friction, the temperature of base material is heated to approximately 80% of its melting point, causing it to soften and flow between the workpieces. Ultimately, this results in a solid-state connection [6]. The advantages of FSW are its savings in terms of energy and the fact that it is environmentally friendly. An excellent high-temperature performance is critical to avoid severe wear with FSW tools [109,110,111]. Currently, W-25Re alloy is the most popular high-temperature material used in FSW tools, which has been widely applied to the welding of steel and titanium alloys [6,112,113,114,115]. Iqbal et al. studied the wear mechanisms of W-25Re and W-25Re-3.2HfC. The results showed that the wear mechanism of the W-25Re was abrasive wear. Nevertheless, the wear mechanism of the W-25Re-3.2HfC was adhesive wear, with HfC debris being observed along the friction path [95]. Compared with W-25Re FSW tools, W-20Re-10HfC FSW tools showed less wear. By analyzing the microstructure of the W-20Re-10HfC FSW tools after wear, the failure mechanism was suggested to be intergranular fracture [115]. The high price of Re increases the cost of W-25Re FSW tools. Therefore, decreasing the Re content and maintaining the excellent friction and wear performance of W-Re alloys is critical to expanding their application in high-temperature FSW tools.

### 3.5. Grain Refinement and Its Effect

Grain refinement is one of the methods to improve the strength, ductility and radiation resistance of tungsten alloys [116,117]. Top-down and bottom-up strategies are two strategies usually used to refine the grain size of tungsten alloys [118].

The top-down approach is a method that decreases the grain size of coarse-grained alloy through severe plastic deformation (SPD) [119]. SPD methods include equal channel angular extrusion (ECAE) [120], accumulative roll bonding (ARB) [121,122], high-pressure torsion (HPT) [123], etc. Faleschini et al. analyzed the fracture behavior of tungsten alloys prepared by different preparation methods. The results indicated that the grain size of tungsten alloys prepared by the high-pressure torsion method was about 300 nm, and they exhibited excellent fracture toughness at room temperature [124]. Kecskes et al. prepared ultrafine-grained tungsten by equal channel angular extrusion (ECAE) and hot rolling. Then, nanocrystalline tungsten was obtained by high-pressure torsion [118]. The results showed that the ultrafine-grained and nanocrystalline tungsten exhibited a higher strength under quasi-static compression. The deformation mechanism of the ultrafine-grained and nanocrystalline tungsten changed from uniform deformation to localized shear when under dynamic compression, which was different from the brittle fracture mechanism of coarse-grained tungsten. Meanwhile, flow softening was observed in the ultrafine-grained and nanocrystalline tungsten during deformation [118]. However, it is unclear whether the improvement in the ductility of the tungsten alloys was merely provided by the grain refinement or the synergies of the grain refinement and high dislocation density generated by severe plastic deformation.

**Figure 6:**
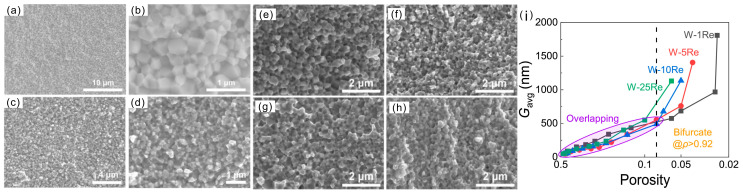
Fracture surfaces of (**a**,**b**) as-sieved W powder with two-step sintering at 1230 °C for 0 h and then at 1180 °C for 10 h [129], (**c**,**d**) as-sieved W-10 powder with two-step sintering at 1200 °C for 0 h and then at 1150 °C for 10 h [116], (**e**) W-1Re powder with two-step sintering at 1150 °C for 0 h and then at 1100 °C for 10 h, (**f**) W-5Re powder with two-step sintering at 1200 °C for 0 h and then at 1150 °C for 10 h, (**g**) W-10Re powder with two-step sintering at 1250 °C for 0 h and then at 1200 °C for 10 h, and (**h**) W-25Re powder with two-step sintering at 1300 °C for 0 h and then at 1250 °C for 10 h [36]. (**i**) The relationship between porosity and average grain size of W-Re alloys with different Re contents [36].

The bottom-up approach involves preparing small-sized raw material powders and obtaining fine-grained samples by using traditional powder metallurgy processes. The ultrafine-grained and nanocrystalline tungsten alloys prepared by this method possess a higher strength than coarse-grained tungsten alloys. However, ultrafine grains introduce various defects such as porosity, weak particle bonding and impurity contamination, which lower the ductility of these alloys [125,126,127,128]. Li et al. used ammonium paratungstate hydrate as a raw material to prepare nano-sized tungsten powder via solution combustion synthesis and hydrogen reduction [36,116,129]. The pressed green compacts were initially sintered under a higher temperature, and all the pores inside the green body entered a thermodynamically sinterable state. Then, it was immediately cooled down to low temperature and kept at a low temperature for a longer period to achieve densification and slow down the grain growth. For instance, the green body was initially heated to the temperatures of 1150, 1200, 1230, 1250 or 1300 °C with no holding time. Following this, the temperature decreased to 1100, 1150, 1180, 1200 or 1250 °C and was maintained for 10 h. The relative density, grain size and hardness of the final obtained W alloy were 99.3%, 290 nm and 7.8 GPa, respectively. The fracture surfaces of the samples are depicted in Figure 6a,b. Que et al. prepared an ultrafine-grained W-10Re alloy at 1200 °C, the relative density, grain size and hardness of which were 98.4%, 260 nm and 6.3 GPa, respectively. The fracture surfaces of the W-Re alloys are shown in Figure 6c,d [116]. Lastly, Que fabricated W-Re alloys with Re contents ranging from 1 to 25 wt.%. The fracture surfaces of the samples are depicted in Figure 6e–h [36]. It was found that the Re accelerated the grain growth. Rapid grain growth in the W-1Re alloy was observed only when the relative density exceeded 97%. The required relative density for rapid grain growth gradually decreased with the increase in the Re content, eventually reaching around 92%, as shown in Figure 6i. It is indicated that W-Re alloys with a high relative density, small grain size and high Re content are difficult to obtain by the bottom-up method. Though this process has achieved synergistic improvements in terms of the relative density and grain refinement, the toughness-related properties have not been reported.

## 4. Conclusions and Perspectives

Tungsten and its alloys have been widely used in high-temperature environments due to their excellent high-temperature performance. However, their low ductility limits their applications. Alloying with Re has been used to improve the ductility of tungsten. The mechanical properties of W-Re alloys can also be significantly improved by solid solution strengthening, second-phase reinforcement and fine-grain strengthening.

(1) The brittleness of tungsten is attributed to its lack of inherent close-packed planes and the weak bonding between its grain boundaries. By adding Re element, the preferred slip plane of tungsten changes from {110} to {112}. Compared with the {110} slip system, the {112} slip system has six more sets of slip planes. Moreover, an appropriate content of Re introduces extra d-valence electrons, thereby softening the tungsten matrix. However, the reasons for the weak bonding between the grain boundaries in tungsten deserves more attention.
(2) Transition elements such as La and Cu and refractory metals such as Ta and Mo can be used for the solid solution strengthening of W-Re alloys. Moreover, transition elements act as activators to lower the sintering temperature of W-Re alloys and inhibit grain growth. Refractory metal elements can inhibit grain growth during sintering. In addition, the alloying order of solid solution elements is also critical to the properties of W-Re alloys. The short-range ordered (SRO) structure has been proven by DFT to enhance the toughness of W-Re-Mo alloys.
(3) Second phases such as HfC and ZrC can be used to enhance the high-temperature strength of W-Re alloys, which exhibit superior creep strength, tensile strength and thermal shock resistance at high temperatures. The strengthening of the performance of W-Re alloys by carbides originates from the interaction between carbides and dislocations as well as their synergistic effect with Re. However, most of the current research has focused on the former, while there is little research on the interaction between carbides and Re. Though the melting point of SiC is lower than that of HfC or ZrC, SiC is also a potential second-phase candidate that can be used to improve the high-temperature properties of W-Re alloys. It can form a toughening phase, W_5_Si_3_, in situ, which enhances the strength of W. However, further exploration is needed regarding the high-temperature performance of SiC-reinforced W-Re alloys.
(4) W-Re alloys with a high dislocation density and fine grain size can be fabricated by using severe plastic deformation (SPD) methods. Ultrafine-grained and nanocrystalline tungsten prepared by the SPD method exhibit a higher strength than coarse-grained tungsten. As the grain size decreases, the deformation mechanism of tungsten changes from uniform deformation to localized shear. Concurrently, flow softening is observed during the deformation process. However, the toughness-enhancing mechanism of the SPD method is yet unclear.

In summary, to advance the development of W-Re alloys, further investigations can be conducted in the following aspects: Utilize advanced equipment to determine the synergistic strengthening mechanism of Re and carbides on W alloys at the atomic scale. Perform atomic-scale tensile experiments to reveal the specific mechanisms of the brittle bonding force between tungsten grains. Explore the mechanism of the in situ synthesis of carbides to control the content of formed carbides and avoid the appearance of large-sized oxides. Investigate the interaction between carbides and the interface of W-Re alloys and clarify their impact on the high-temperature deformation and strengthening behavior of W-Re alloys.

## Data Availability

Not applicable.
